# Clinical outcome of transurethral enucleation of the prostate using the 120-W thulium Laser (Vela™ XL) compared to bipolar transurethral resection of the prostate (TURP) in aging male

**DOI:** 10.18632/aging.102720

**Published:** 2020-01-28

**Authors:** Chen-Pang Hou, Yu-Hsiang Lin, Horng-Heng Juang, Phei-Lang Chang, Chien-lun Chen, Pei-Shan Yang, Ke-Hung Tsui

**Affiliations:** 1 Department of Urology, Chang Gung Memorial Hospital at Linkou, Taiwan, Republic of China; 2 School of Medicine, Chang Gung University, Taiwan, Republic of China; 3 Graduate Institute of Clinical Medical Sciences, College of Medicine, Chang Gung University, Taiwan, Republic of China; 4 Department of Anatomy, School of Medicine, Chang Gung University, Kwei-Shan, Tao-Yuan, Taiwan, Republic of China

**Keywords:** benign prostate hyperplasia, bladder outlet obstruction, aging, laser

## Abstract

This study compared the surgical outcomes of the 120-W Thulium laser (Vela™ XL) enucleation of the prostate and bipolar transurethral resection of the prostate (TURP) in terms of efficacy, safety, and improvements of quality of life (QoL) in patients with benign prostate hyperplasia (BPH). Records were obtained from January 2014 to September 2018 for selected patients with symptomatic BPH who underwent 120-W Thulium laser (Vela™XL) prostate enucleation and bipolar TURP in our institution. All the patients selected met the surgical criteria for TURP and had received medical treatment for at least 3 months. Patients were excluded if their ECOG performance status was >1, if they had active malignant disease, of if they had a history of prostate surgery or reconstruction surgery of the urinary system. Patients decided which treatment option would be performed. Both the procedures were conducted by a single surgeon. Clinical outcomes such as changes in the International Prostate Symptom Score (IPSS) score, urodynamic parameters, drug consumption, pain scores, and QoL were evaluated. The rate of urinary tract infection, recatheterization, additional analgesic requirement, return to the emergency department for treatment, and other surgical complications was analyzed and compared between the two cohorts. A total of 276 patients met the inclusion criteria. Among them, 141 patients received bipolar TURP, where as 135 decided to receive laser vaporesection. No significant difference was observed in age, PSA level, prostate volume, and comorbidities between the two cohorts. Pre-operative (pre-op) urodynamic parameters were also identical, except that the laser surgery group had a higher rate of admission with a urinary catheter (24.4% *vs*. 14.2%, *p*=0.044). The operating time was longer in the laser surgery group (79.3 minutes *vs.* 62.4 minutes, *p*<0.001). However, enucleation using the Thulium laser was superior to bipolar TURP in terms of post-operative (post-op) pain status, including the numeric rating scale of pain, rate of additional narcotic use, and oral analgesic requirement. Compared with bipolar TURP, laser enucleation achieved a higher improvement in the QoL score at post-op follow-up at 2 weeks and 3 months. Nevertheless, the complication rate, changes in IPSS score, Qmax, and post-op medication-free survival were statistically identical in the two cohorts. Our data revealed that compared with bipolar TURP, 120-W Thulium laser (Vela™ XL) enucleation of the prostate achieved lower post-op pain and higher improvement in the short-term QoL of patients after surgery.

## INTRODUCTION

Benign prostatic hyperplasia (BPH) is a major cause of lower urinary tract symptoms (LUTSs) in the aging male population, and it affects approximately 210 million men globally [1]. In addition, the prevalence of BPH/LUTS is expected to increase sharply in the coming decades [2]. The symptoms of BPH include decreased urinary flow and advancing voiding and storage symptoms that result in acute or chronic urinary retention (UR) [3]. Moderate to severe LUTS also significantly affects all quality of life (QoL) parameters for aging men [4]. Both α1-blockers and transurethral resection of the prostate (TURP) achieve favorable outcomes in most patients with benign prostate obstruction (BPO) [5]. Although medical treatment is available for BPO, surgical intervention is an appropriate option for patients with moderate to severe LUTS and for patients who have developed acute UR or other BPH-related complications [6]. Although TURP remains the dominant and definitive treatment option for BPH/BPO [7], it involves potential morbidities, including urinary tract infection(UTI) (1.7%–8.2%), UR (3%–9%), hematuria with clot retention (2%–5%), urethral strictures (2.2%–9.8%), and bladder neck contractures (0.3%–9.2%) [8]. As a result, a variety of laser systems and techniques for treating BPH/BPO have been introduced to overcome the aforementioned problems, with the aims of lower blood loss, clearer vision of the surgical field, shorter catheterization time, and lower morbidity [9]. The high-power continuous-wave Thulium laser was first introduced in 2005 for treating BPH/BPO [10]. According to the latest guidelines, Thulium laser enucleation of the prostate (ThuLEP) is recommended as an appropriate and prostate size–independent alternative to resolve BPH/BPO [11]. In this study, we conducted a head-to-head comparison of the surgical outcomes of the 120-W Thulium laser (Vela™ XL) enucleation of the prostate with those of the bipolar resection of the prostate in terms of efficacy, safety, and improvement of life quality in patients with symptomatic BPH/BPO.

## RESULTS

**Figure 1:**
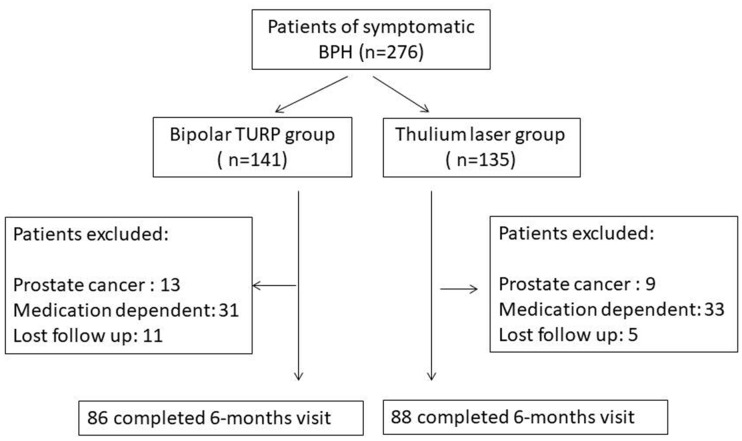
**Flow chart of patient treatment.**

**Figure 2:**
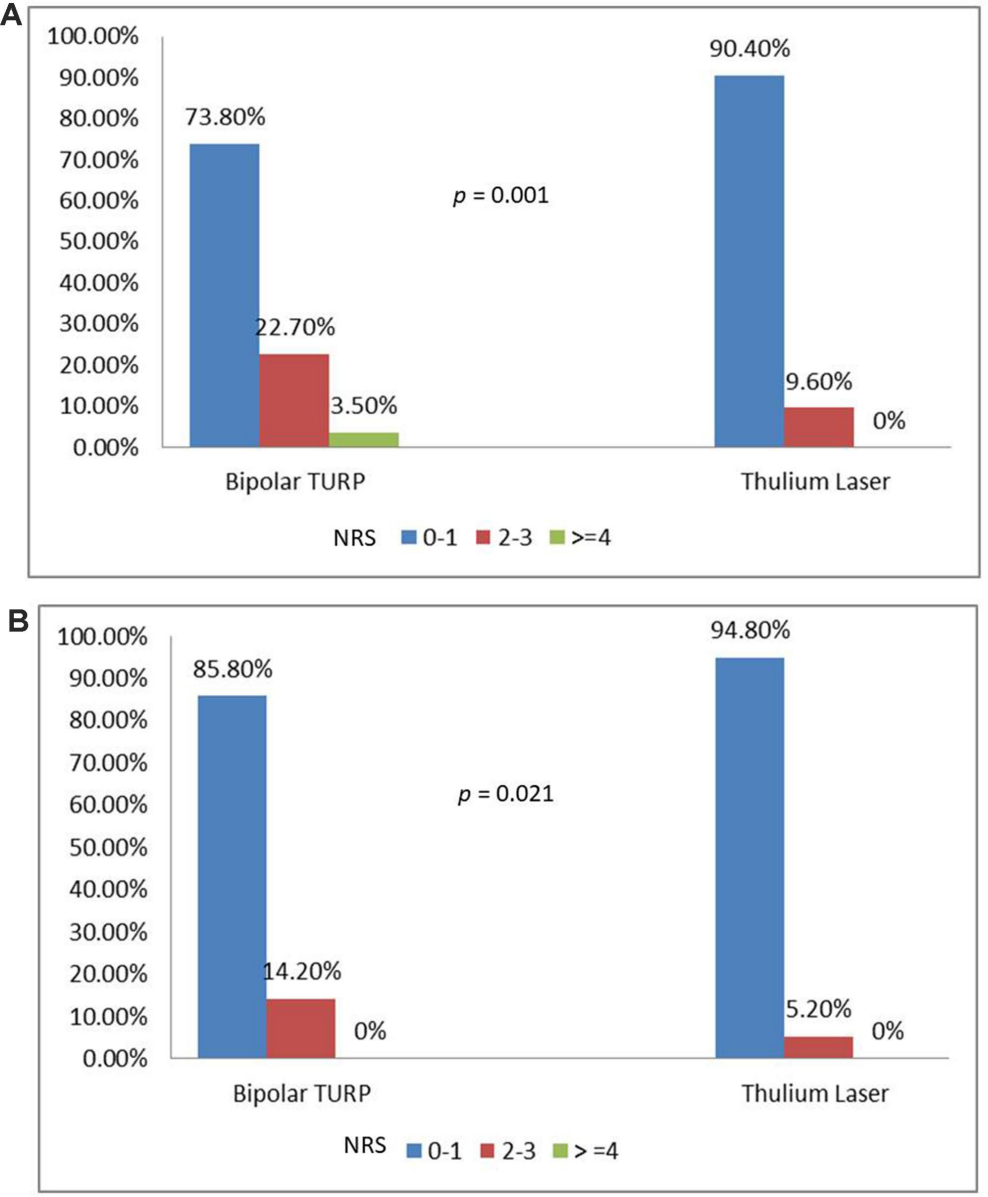
(**A**) Numeric rating scale on post-op Day 1. (**B**) Numeric rating scale on post-op Day 2.

The flow chart of patient treatment is illustrated in Figure 1. Of the 276 patients who met the inclusion criteria, 141 patients received bipolar TURP and 135 patients received ThuLEP. The baseline characteristics of patient’s are presented in Table 1. The two groups were identical in terms of age, PSA, renal function, prostate volume, and co-morbidities. The pre-operative (pre-op) urinary conditions of the two groups are presented in Table 2. No statistically significant difference was observed in the initial IPSS score (either voiding or storage), QoL, Qmax, and PVR between the two groups. No significant difference was observed in the type and duration of urological medication consumption between the two groups. In total, 23% of patients in the TURP group and 33% of patients in the ThuLEP group claimed that they had never gone to a medical institution for catheterization because of UR, and the ratio was statistically identical between the two groups. Notably, a higher proportion of patients in the ThuLEP group was hospitalized with urinary catheters compared with the proportion in the TURP group (24.4% *vs.*14.2%, *p*=0.044). The intra- and perioperative data are depicted in Table 3. TURP required a shorter operating time (62.4 ± 26.3 minutes *vs*. 79.3 ± 27.2 minutes, *p*< 0.001) compared with ThuLEP. The NRS on post-op Day 1 and Day 2, as illustrated in Figure 2A and 2B, revealed that ThuLEP was superior to TURP in terms of post-op pain. In addition, compared with the ThuLEP group, a higher proportion of patients in the TURP group required an additional injection of narcotics after surgery (20.6% *vs*. 5.2%, *p*<0.001). In addition, compared with the ThuLEP group, a higher proportion of patients in the TURP group required oral analgesics for more than 1week after surgery (12.2% *vs*. 4.4%, *p*=0.039). Nevertheless, no statistically significant difference was observed in the length of hospital stay, percentage of tissue removed, and re-catheterization rate within 1 month post operation. No blood transfusion was required in either group. The majority of patients experienced no complication or they experienced grade I–II complications. Particularly, compared with the TURP group, a higher proportion of patients in the ThuLEP group returned to the emergency department within 1monthpost-op, although this was not statistically significant (11.9% *vs.* 5.0%, *p*=0.064). Delayed prostate bleeding was the most common event reported among these patients (7 of 16).

**Table 1:** Baseline characteristics of patients.

**Parameter**	**Bipolar TURP (n=141)**	**Thulium laser (n=135)**	***p* value**
Age (mean+SD)	68+9.4	70+9.1	0.282
PSA (μg/l)	4.8+5.0	5.7+5.6	0.246
Cr (mg/dl)	1.0+0.4	0.9+0.4	0.866
Prostate volume (ml)	48.4+11.6	53.3+14.5	0.096
Comorbidities (n, %)			0.825
DM	23 (16.3%)	32 (23.7%)	
HTN	59 (41.8%)	65 (48.1%)	
CAD	6 (4.3%)	9 (6.7%)	
Arrhythmia	6 (4.3%)	7 (5.2%)	
Stroke	7 (5.0%)	13 (9.6%)	
CRI	11 (7.8%)	9 (6.7%)	

Abbreviations: TURP: transurethral resection of the prostate; SD: standard deviation; PSA: prostate-specific antigen; Cr: creatinine; DM: diabetes mellitus; HTN: hypertension; CAD: coronary arterydisease; CRI: chronic renal insufficiency.

**Table 2:** Pre-op urinary condition of patients.

**Parameter**	**Bipolar TURP**	**Thulium laser**	***p* value**
IPSS (total)	24.6 + 4.5	25.5 + 3.8	0.148
IPSS (voiding)	15.2 + 3.2	15.7 + 2.9	0.166
IPSS (storage)	9.5 + 3.1	9.7 + 3.0	0.608
IPSS (QoL)	4.6 + 0.6	4.8 + 0.6	0.072
Qmax (ml/s)	10.0 +8.4	7.8 + 5.1	0.065
PVR (ml)	118.0 + 140.5	125.6 + 137.1	0.678
Medication (n, %)			
α-blockers	141 (100%)	135 (100%)	1.000
Anti-muscarinics	21 (14.9%)	20 (14.8%)	0.985
Bethanecol	23 (16.3%)	23 (17.0%)	0.795
Duration of medication (medium, month)	5 (3-120)	4 (3-120 )	0.968
Ever UR. (n, %)	33 (23.4%)	45 (33.3%)	0.089
Admitted with a catheter (n, %)	20 (14.2%)	33 (24.4%)	0.044*

Abbreviations: TURP: transurethral resection of the prostate; IPSS: International Prostate Symptom Score; QoL: quality of life; Qmax: maximum flow rate; PVR: post-void residual urine; UR: urinary retention.

**Table 3:** Intra- and perioperative data.

**Parameter**	**Bipolar TURP**	**Thulium laser**	***p* value**
OP time (min)	62.4 + 26.3	79.3 + 27.2	<0.001 *
Hospitalization duration (days)	4.3 + 1.7	4.2 +1.5	0.201
Percentage of tissue removed (%)	47.6 + 13.1	46.9 +12.4	0.612
Blood transfusion (n, %)	0	0	
Re-catheterization within 1 month (n, %)	14 (9.9%)	16 (11.9%)	0.700
Additional narcotic use (n, %)	29 (20.6%))	7 (5.2%)	0.003*
Analgesic requirement>1 week (n, %)	17 (12.1%)	6 (4.4%)	0.039 *
Returned to ER within 1 month (n, %)	7 (5.0%)	16 (11.9%)	0.064
	UR: 4	UR: 2	
	UTI:2	UTI: 4	
	Epididymitis : 1	AGE : 1	
		Delay hematuria: 7	
		Stroke 1	
		Pneumonia: 1	

Abbreviations: TURP: transurethral resection of the prostate; OP: operation; UR: urinary retention; UTI: urinary tract infection; AGE: acute gastroenteritis

**Figure 3:**
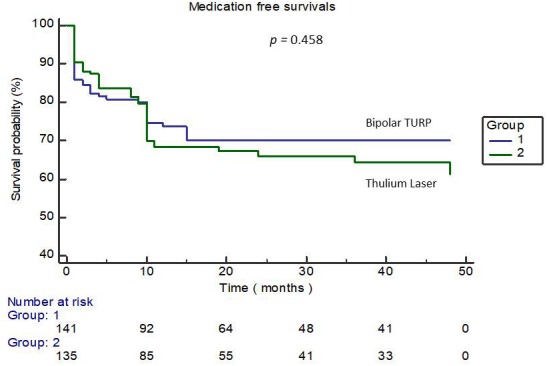
**Kaplan–Meier curve-illustrating the medication-free survival rates of the two groups.**

Eighty-six patients of the TURP group and 88 of the ThuLEP group completed this tracking program for at least 6 months, as shown in figure 1. Both groups achieved favorable surgical outcomes in terms of Qmax, IPSS score, and QoL score at post-op 2 weeks, 3 months, and 6 months, as presented in Table 4. However, the ThuLEP group showed superior outcomes to the TURP group at post-op 2 weeks regarding the changes in the IPSS voiding score (−12.4 ± 3.7*vs.*−10.8 ± 4.4, *p*=0.003) as well as changes in the QoL score (−3.1 ± 0.8*vs.*−2.8 ± 0.9, *p*=0.006). The outcomes in the ThuLEP group remained superior to those of the TURP group regarding changes in the QoL score (−3.4 ± 0.9*vs*.−3.1 ± 0.9, *p*=0.045) at 3 months post-op. Nevertheless, during follow-up at 6 months after surgery, all the indicators of the two groups were statistically identical, suggesting that the efficacy of the two techniques was identical at 6 months post-op. Given that the optimal results of surgical treatment for BPH/BPO are completely independent from urological medication, the medication-free survival rates were evaluated between the two groups, as illustrated in Figure 3. Kaplan–Meier curves revealed that approximately70% of patients in our study did not need any urological medication (including α-blockers, antimuscarinics, β3 agonists, bethanechol, and DDAVP) within 2 years post-op. The medication-free survival rates of both groups were also statistically identical (*p*=0.458).A total of eight patients (6 in the TURP groups and 2 in the ThuLEP group) in our study developed bladder neck contracture before the end of follow-up, with a mean time-to-contracture of 6.7 months.

**Table 4:** Questionnaire on functional changes and quality of life.

	**Bipolar TURP**	**Thulium Laser**	***p* value**
Post-OP 2 weeks			
Δ Qmax (ml/s)	4.8 + 12.3	5.9 + 8.6	0.419
Δ IPSS ( voiding )	-10.8 +4.4	-12.4 +3.7	0.003*
Δ IPSS ( storage)	-7.6 +3.2	-7.9 +3.0	0.371
Δ IPSS (QoL)	-2.8 + 0.9	-3.1 +0.8	0.006*
Post-OP 3 months			
Δ Qmax (ml/s)	7.1 +11.5	9.4 +6.6	0.053
Δ IPSS ( voiding )	-13.4 +3.1	-14.1 +3.0	0.095
Δ IPSS ( storage)	-7.3 +3.1	-7.7 + 3.3	0.352
Δ IPSS (QoL)	-3.1 +0.9	-3.4 +0.9	0.045*
Post-OP 6 months			
Δ Qmax (ml/s)	7.5 + 5.9	8.1 + 5.7	0.364
Δ IPSS ( voiding )	-13.4 +3.1	-14.1 +3.0	0.110
Δ IPSS ( storage)	-7.9 +3.3	-8.1 +3.10	0.656
Δ IPSS (QoL)	-3.3 +0.8	-3.5 +0.8	0.127

Abbreviations: TURP: transurethral resection of the prostate; IPSS: International Prostate Symptom Score; QoL: quality of life; Qmax: maximum flow rate; UR: urinary retention

## DISCUSSION

Although TURP has been the gold standard of surgical intervention for BPH/BOO, it is associated with potential surgical risks [8]. To minimize the risks caused by TURP, PKRP has been developed and is regarded as a safe and effective therapy for the surgical management of symptomatic BPH [12]. A systematic review revealed that although the efficacy was identical for both methods, bipolar TURP had more favorable outcomes than monopolar TURP in terms of the safety profile [13]. In addition to TURP, several laser devices have been developed. Among them, green light vaporization as well as laser enucleation with holmium are the two most intensively investigated and valid clinical options currently [14]. Moreover, the Thulium laser, possessing 2013-nm wavelength and 0.2-mm penetration depth, takes water as the chromophore that absorbs, and energy is released by a visible continuous wave [9]. In clinical practice, two types of thulium lasers are available, namely the Tm-YAG (Revolix) and Tm-fiber (Vela™ XL) [15]. In our study, the Tm-fiber (Vela™ XL) laser was used to conduct ThuLEP. The technique for conducting ThuLEP was first presented by Bach et al. [16]. It is a new type of surgical treatment that has been recently implemented, and it has demonstrated stability in improving Qmax and QoL and reducing IPSS scores [15]. ThuLEP incorporates a Thulium laser and blunt enucleation with the resectoscope sheath to perform an apical incision of the prostatic tissue down to the capsule [27]. The prostate tissue is enbloc enucleated and pushed forward to the bladder before being grinded with a morcellator to obtain the specimen. Compared with TURP, ThuLEP is more beneficial in terms of minimal blood loss and higher intraoperative safety, lower normal saline irrigation, shorter catheterization, and shorter hospital stay. However, it requires a longer operating time [17]. Regarding safety concerns, Tal et al. disclosed that compared with TURP, ThuLEP presents decreased risks of TUR syndrome development, blood transfusion, and urethral stricture [18]. Other complications reported include recatheterization, temporary urinary incontinence, UTI, and retrograde ejaculation, but the rates of occurrence of these complications are similar to TURP [18]. Our research demonstrates that bipolar TURP and ThuLEP have similar therapeutic effects in terms of the improvement of Qmax, IPSS score, and PVR. Our findings are similar to previous findings. However, in our study, the probability of complications in both groups was comparable. This may be because all the operations were performed by a single experienced surgeon, and our patients had been carefully selected based on surgical indications and general performance status.

The main difference between our research and previous research is that we further analyzed patients’ post-op pain score, changes in the QoL score, and records of analgesic use. In our daily practice, we have observed that compared with conventional TURP, patients who undergo ThuLEP appear to experience less pain. Our research validates this hypothesis. Our study revealed that ThuLEP is superior to bipolar TURP in terms of post-op pain scores, injection of narcotics after surgery, and requirement of oral analgesics. We believe that the depth of the thermal penetration is the main factor. The absorbed energy of the Thulium laser at the tissue surface leads to instant vaporization and limits the penetration depth for approximately0.2mm [19]. In addition, the penetrating thermal depth of bipolar TURP is significantly higher than that of the Thulium laser. Maddox et al. revealed that the mean depth of thermal injuries was 2.4±0.84mm (range: 0.3–3.5 mm) [20]. Bipolar TURP causes deeper thermal injuries than ThuLEP, and as a result, it causes more post-op pain. Notably, 16 (11.9%) patients in the ThuLEP group returned to the emergency department for treatment 1 month post-op, whereas only 7 (5%) patients in the TURP group returned to the emergency department. Although the reasons for returning to the emergency department were not all related to surgery, seven people in the laser group returned to the emergency department because of delayed hematuria. By contrast, none of the patients in the TURP group returned to the emergency department for that reason. All patients were managed with conservative treatment and did not require transurethral coagulation surgery. Some studies have reported that delayed bleeding is a noteworthy issue in prostate surgery using the Thulium laser, and we believe it may be caused by the shallow thermal depth of energy. Chuang et al. reported delayed bleeding in 19 of 150 patients (12.6%) who underwent prostate Thulium laser treatment, and 4 of them required transurethral coagulation under general anesthesia [21].

Another issue of interest in this study is urological medication withdrawal after surgery. The ultimate goal of undergoing surgery for patients with BPH/BPO is that the patients should become medication-free in the future. According to Han et al., numerous patients have persistent voiding dysfunction and rely on medication after surgical treatment for LUTS/BPH. Older age, a history of diabetes or stroke, and pre-op use of antimuscarinics are potential risk factors [22]. Our study revealed that approximately70% of patients in both groups remained independent of urological medications at 2 years post-op, and this curve became a plateau phase after this time point. In addition, the medication-free survival rates of both groups were not significantly different, indicating that the treatment efficacy of both groups was identical in the long term.

This study has limitations due to their search design. First, this prospective study was not randomized in terms of the patient group. Patients were free to choose their operation method. Nevertheless, the baseline characteristics and pre-op urinary function of patients were grossly identical between the two groups. Therefore, it may not cause too much of a bias in the analysis. Second, a pressure flow urodynamic study [23] was not conducted inpatients before they underwent surgery. Although the pressure flow urodynamic study remains the gold standard for diagnosing BOO and provides more information on urinary function, this is an unpleasant and invasive examination for patients and is not included in our daily practice. However, we believe that our study is innovative and valid because it is a head-to-head comparison of two commonly used prostate surgical techniques in terms of efficacy, safety, post-op pain, and improvement of life quality. Our findings revealed that enucleation of the prostate using the 120-W Thulium laser yielded lower post-op pain and higher improvement of the short-term QoL. Since postoperative pain in elderly patients has a high correlation between adherence to treatment guidelines and patient satisfaction [24], we believe that prostate surgery using a ThuLEP technique is worth considering for older patients.

## CONCLUSIONS

Both bipolar TURP and ThuLEP are effective and safe procedures for the treatment of BPH/BPO. However, compared with bipolar TURP, enucleation of the prostate using the 120-W Thulium laser yields lower post-op pain and higher improvement of the short-term QoL after surgery and as a result a worthwhile choice for older patients.

## MATERIALS AND METHODS

### Patients

Records were obtained from January 2014 to September 2018 for selected patients with symptomatic BPH who underwent 120-W Thulium laser (Vela™ XL) prostate enucleation or bipolar TURP in Urology department, Chang-Gung Memorial Hospital, Linkou, Taiwan, following the institutional review board approval. Both procedures were conducted by a single skilled surgeon. Patients were free to choose the operation method, and they signed consent forms. Before surgery was performed, all individuals underwent comprehensive assessment, including medical history interview, physical examination, digital rectal examination (DRE), International Prostate Symptom Score (IPSS), QoL score, serum prostate-specific antigen (PSA), transrectal ultrasound (TRUS), post-void residual urine volume (PVR),and peak flow rate (Qmax). Patients received TRUS biopsy if an abnormality was detected during the DRE for excluding prostate cancer. The inclusion criteria were as follows: age <80 years, IPSS ≥20,Qmax ≤15 mL/s, and prostate volume>30 g. All individuals met TURP surgical indications [25] and had received medical therapy for at least3 months prior to surgery. Patients were excluded if their ECOG performance status was >1, if they had active malignant disease, or if they had a history of prostate surgery or reconstruction surgery of the urinary system. Patients with neurogenic bladder or LUTS resulting from reasons other than BPH were also excluded.

### Equipment and surgical techniques used

All operations in the laser surgery group were conducted using a120-W Thulium laser (Vela™ XL, Boston Scientific, Marlborough, Massachusetts, USA) with a continuous wavelength of 1.94μm. The energies used for enucleation and resection were60and 120W, respectively. The laser fiber was a Light Trail Single-Use Laser Fiber with a wavelength of 600μm. An Olympus 26F continuous-flow resectoscope was used to introduce the laser fiber. Irrigation was used in all processes with a 0.9% sodium chloride solution. The Wolf Piranha Morcellator was used to grind the enucleated prostate tissue. Operations in the PKRP group were conducted using the Olympus SurgMasterUES-40 bipolar generator and the OES-Pro bipolar resectoscope (Olympus Europe, Hamburg, Germany). The standard settings of energy were 200 and 120W for cutting and coagulation, respectively. During surgery, all patients were placed in the lithotomy position, and whether general or spinal anesthesia should be performed was decided by the on-duty anesthesiologists. The operation for the TURP group was performed using the conventional TURP technique [26], whereas the technique used in the ThuLEP group was the one described by Herrmann [27]. To provide intermittent and permanent irrigation, a three-way Foley catheter (22 Fr) was placed in the bladder at the end of both processes. Hemostasis using catheter balloon traction to compress the prostate was not conducted in both groups. The catheters were scheduled to be removed on post-operative (post-op) Day 2 in both groups. Prophylactic and post-op antibiotics usage followed the quid-line recommendations [28]. Once a patient demonstrated signs of infection post-operation, suitable antibiotics were used based on the bacterial culture and drug sensitivity outcomes. Post-op pain was evaluated using the numeric rating scale (NRS) on post-op Day 1 and Day 2. The standard analgesic regimen for both procedures was 7 days of acetaminophen. If patients still felt pain despite the consumption of acetaminophen, they could ask for an injection of opioids analgesic (nalbuphine, 10mg, intravenously). Regardless of the combination of urological medication pre-op, 0.4mg Ocas once daily was prescribed to all patients for only 1week, and they were evaluated under a medication-free status. During follow-up, the physician decided whether to re-medicate the patient according to the patient’s condition.

### Outcome evaluation and follow-up

**Figure 4:**
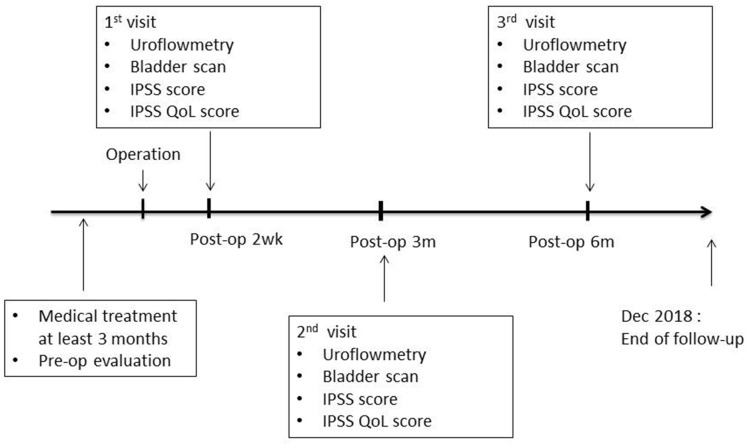
**Study flow diagram.**

Perioperative outcomes were recorded, including the operating time, surgical complications, analgesic consumption, NRS score of pain [29], post-operative re-catheterization, and hospital stay length. At 2 weeks and 3 and 6 months after surgery, patients returned for a follow-up visit. During the visits, the IPSS score, QoL, Qmax, PVR, and rate of urological medication continuity were evaluated, and the incidence of complications was recorded. If patients had any problems after 6 months of follow-up, they were asked to return to the clinic for evaluation and treatment. The study flow diagram is illustrated in Figure 4.

### Statistical analysis

MedCalc version 16.2.1 for Windows (MedCalc Software bvba, Ostend, Belgium) was used for statistical analysis. All parameters are presented as mean (or median) ± standard deviation. The chi-square test was used for analyzing qualitative variables, whereas the Student *t* test was used for analyzing quantitative variables; *p*< 0.05 was considered statistically significant.
